# Quantitative Evaluation of the 3D Anatomy of the Human Osseous Spiral Lamina Using MicroCT

**DOI:** 10.1007/s10162-023-00904-3

**Published:** 2023-07-05

**Authors:** Gabriela O. Bom Braga, Annapaola Parrilli, Robert Zboray, Milica Bulatović, Franca Wagner

**Affiliations:** 1 ARTORG Center for Biomedical Engineering Research, University of Bern, Bern, Switzerland; 2 Center for X-Ray Analytics, Empa - Swiss Federal Laboratories for Materials Science and Technology, Überlandstrasse 129, 8600 Dübendorf, Switzerland; 3 Institute of Diagnostic and Interventional Neuroradiology, Inselspital, Bern University Hospital, University of Bern, Bern, Switzerland

**Keywords:** Osseous spiral lamina, OSL thickness and width, Bony pillars, Porosity, microCT, Human

## Abstract

**Purpose:**

The osseous spiral lamina (OSL) is an inner cochlear bony structure that projects from the modiolus from base to apex, separating the cochlear canal into the scala vestibuli and scala tympani. The porosity of the OSL has recently attracted the attention of scientists due to its potential impact on the overall sound transduction. The bony pillars between the vestibular and tympanic plates of the OSL are not always visible in conventional histopathological studies, so imaging of such structures is usually lacking or incomplete. With this pilot study, we aimed, for the first time, to anatomically demonstrate the OSL in great detail and in 3D.

**Methods:**

We measured width, thickness, and porosity of the human OSL by microCT using increasing nominal resolutions up to 2.5-µm voxel size. Additionally, 3D models of the individual plates at the basal and middle turns and the apex were created from the CT datasets.

**Results:**

We found a constant presence of porosity in both tympanic plate and vestibular plate from basal turn to the apex. The tympanic plate appears to be more porous than vestibular plate in the basal and middle turns, while it is less porous in the apex. Furthermore, the 3D reconstruction allowed the bony pillars that lie between the OSL plates to be observed in great detail.

**Conclusion:**

By enhancing our comprehension of the OSL, we can advance our comprehension of hearing mechanisms and enhance the accuracy and effectiveness of cochlear models.

## Introduction

The osseous spiral lamina (OSL) is a bony structure inside the cochlea that projects from the modiolus from base to apex, separating the cochlear canal into the scala vestibuli and the scala tympani. Despite being described as early as 1851 [1], misconceptions regarding its anatomy [2–4] and mechanical behavior are only now being corrected. The advent of technologies such as scanning electron microscopy combined with 3D reconstructions has improved the understanding and visualization of inner ear anatomy. Additionally, recent findings suggesting a cribriform structure of the OSL plates gave rise to the hypothesis that its mobility may influence air and bone conductive hearing [5–7]. As anatomically revealing as scanning electron microscopy is, the need for tissue preparation, potential for introduction of artifacts, and typical anisotropic resolution (0.5 × 0.5 × 100 µm) make this technique suboptimal for cochlear investigation.

To overcome these limitations, non-destructive imaging modalities based on computed tomography and magnetic resonance are well-established to meet the varying levels of resolution required for pre-clinical and clinical applications. For instance, clinical computed tomography is available with an isotropic resolution of 500 µm or less and magnetic resonance of 300 µm at 7 Tesla. For pre-clinical purposes, these same imaging modalities usually provide isotropic resolution typically at levels of 10 to 100 µm [8–10]. Laboratory-based micro-computed tomography (microCT) is an imaging technique that is increasingly applied for research in biomedical and life sciences. MicroCT was introduced to complement histological evaluations, as a non-destructive, three-dimensional imaging technique at a microscopical level. At the same time, high-intensity synchrotron radiation has enabled better visualization and contrast of the soft structures inside the cochlea [11]. Unfortunately, most microCT studies either use animal models or are restricted to the oval and round windows [11, 12]. Meanwhile, other anatomical structures of cochlea remain subdued [13, 14]. Given the bony morphology of the OSL microCT is therefore well-suited for generating images up to single-digit micron resolutions enabling detailed 3D reconstructions to be created for the first time.

Although inconsistent with the classical concept of a rigid OSL, this structure was described as porous in early anatomical studies, but not visually demonstrated [1–4, 15, 16]. The radial length and porosity can explain the observed movement of the OSL in response to sounds in human cadaveric temporal bones, as described by Raufer et al. [5]. Even though its function remains undetermined, it is known that the OSL can have an impact on sound response and the motion of the cochlear partition (CP) [6, 17]. The higher repercussion relies on bone-conducted sound stimulation. The compression and expansion vibrations of the otic capsule create a vibrational response on the basilar membrane [18], and no difference between air conduction and bone conduction mechanisms was found in experiments using cadaveric fresh-frozen human temporal bones [7]. Bone conduction sound transmission is considered to be based on inertial effects [19, 20]. In this context, the inertial vibration of the OSL may have a partial impact on the overall response of basilar membrane vibrations to sound, especially at high frequencies [7]. All these previous theoretical descriptions corroborate the idea of a flexible OSL. However, the current models of the cochlea assume that the OSL is rigid. The findings presented in this study can be utilized to integrate a flexible OSL into future cochlear models. Moreover, to the best of our knowledge, there has so far been no detailed visualization of its morphological characteristics.

The aim of this pilot study is to present a detailed 3D reconstruction of the osseous spiral lamina, which has not been previously studied in such depth. By gaining a better understanding of the OSL, we can further advance our knowledge of the mechanisms of hearing and increase the reliability and efficiency of cochlear models.

For this purpose, we measured the width and thickness of the human OSL by microCT using increasing nominal resolutions (14.0-, 13.0-, 4.5-, 2.5-µm voxel size).

Additionally, a porosity distribution study of the individual plates at the basal and middle turns and the apex was conducted. Furthermore, the 3D reconstruction allowed the bony pillars (BP) that lie between the OSL plates to be observed in great detail.

## Materials and Methods

### Cochlea Removal

With the permission of the local ethics review board (KEK Bern, Switzerland, Project-ID 2018–00770), cadaveric samples of formalin flushed human cochleae were analyzed in this study (*n* = 3). Specimens were chosen randomly from the whole cephalus specimens available, and the temporal bones were carefully removed (two right side and one left).

**Fig. 1:**
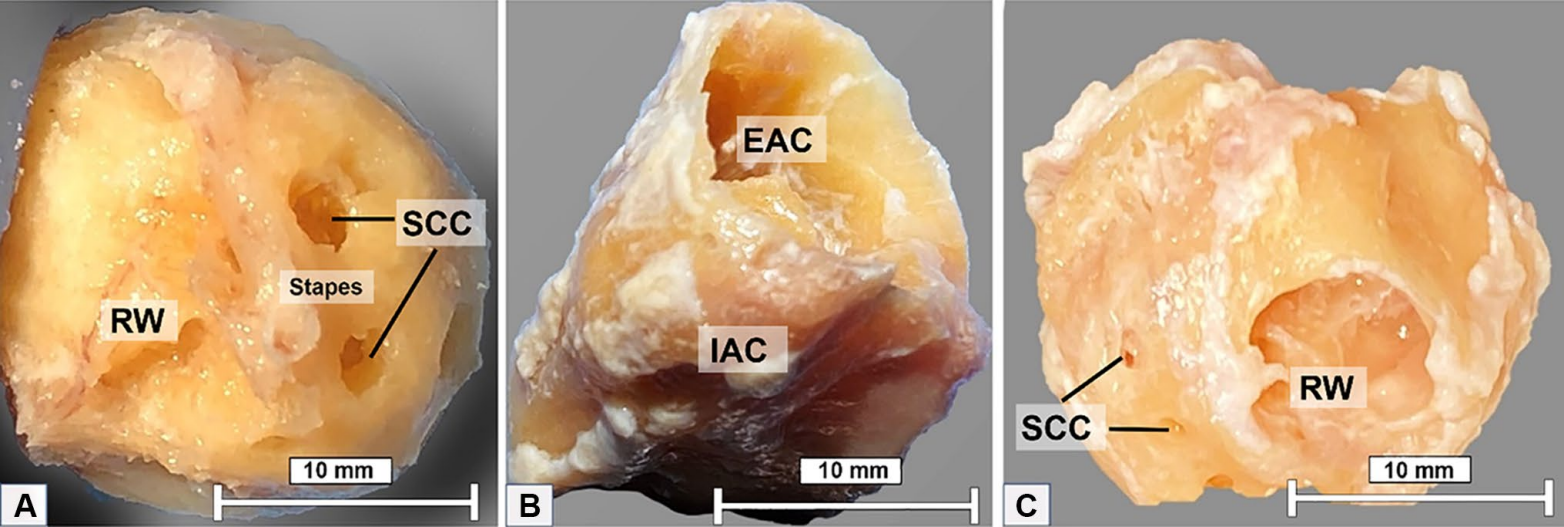
**A** Cochlea 1; **B** cochlea 2; **C** cochlea 3; refinement to 1.5 cm in size for microCT analysis. RW, round window; SCC, semicircular canals; IAC, internal auditory canal; EAC, external auditory canal

Owing to the limitations of the detector size and to avoid excessive parasitic absorption by the surrounding bone structures, the temporal bone surrounding the cochlea had to be removed. The dissection started with middle ear inspection. An artificial titanium stapes prosthesis was observed in cochlea 3 confirming that the patient had a clinical diagnosis of otospongiosis. The other two cochleae showed no signs of surgical procedures or middle or inner ear diseases. The post-auricular incision was made, and the mastoid bone was exposed. Later, a mastoidectomy and subsequent labyrinthectomy, as described by Gulya et al. [21], were performed. These procedures remove the bone surrounding the cochlea, including the semicircular canals and part of the vestibule and middle ear structures, leaving the cochlea exposed. The stapes were kept in place for anatomical reference. The VIIth and VIIIth cranial nerves of the internal auditory canal were detached from the skull base, and the cochlea was removed from its encasement. At this point, the cochlea went through a dissection refinement process until it was no more than 1.5 cm in diameter (Fig. 1).

Since the effects of otosclerosis on the OSL are as yet unknown, cochlea 3 was excluded from this porosity study, having its own description on our recent separate publication [22]. However, since the length, thickness, and width of the cochlea can be expected to be unchanged, these data were included in the study.

### MicroCT

**Fig. 2:**
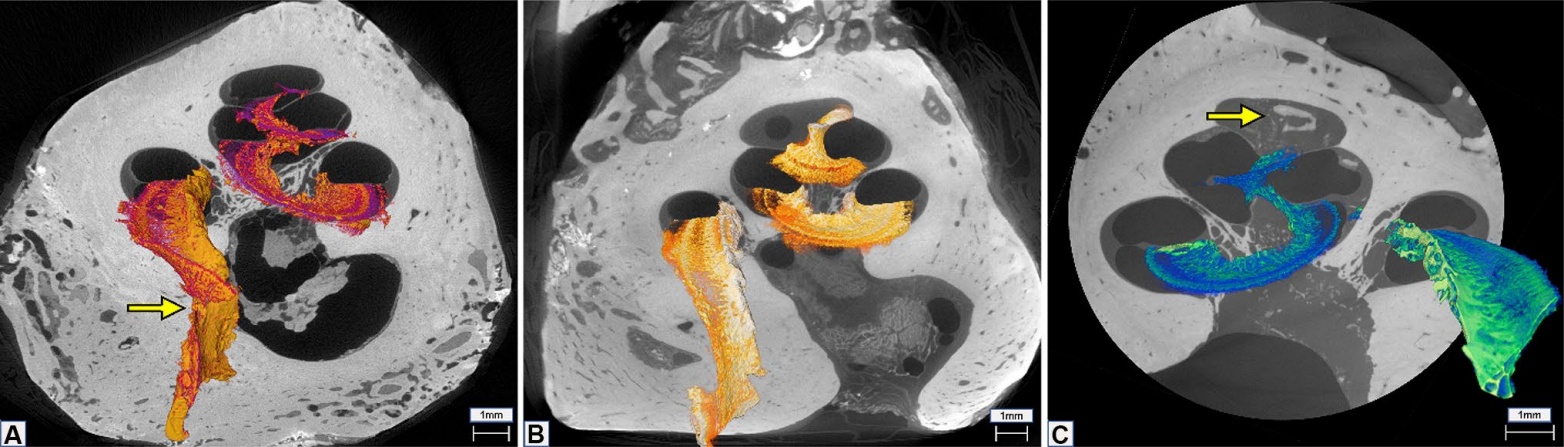
**A** MicroCT of cochlea 1 at 13-µm voxel size resolution. Yellow arrow points to a tear on the OSL. **B** MicroCT of cochlea 2 at 14-µm voxel size resolution. **C** MicroCT at 4.5-µm voxel size resolution. Yellow arrow shows inner cochlear calcifications and fibrotic tissue formation

MicroCT analysis was carried out using an EasyTom XL Ultra 230–160 micro/nanoCT scanner (RX Solutions, Chayanod, France). The scanner features a Hamamatsu nanofocus, transmission X-ray tube with a 1-µm-thick tungsten target on a diamond window. The tube was operated with an LaB6 cathode. The scans were performed using a Varian PaxScan 2520DX detector (flat panel with amorphous silicon and a CsI conversion screen; 1920 × 1536-pixel matrix; pixel pitch of 127 µm^2^; 16 bits of dynamic range). The tube was operated at 70 kV and a current of 60 µA. The full cochlea sample was scanned at a voxel size of 13 and 14 µm (cochleae 1 and 2, respectively) (Fig. 2). As can be seen from Fig. 1, the overall size of the samples harvested for microCT analysis is slightly different both because of normal variability between samples and because of their removal and preparation procedure. Since imaging is performed in cone-beam geometry and the obtainable voxel size is determined by the physical size and number of pixels of the detector and the sample size, to obtain the highest nominal resolution possible with respect to the field of view, the whole cochlea samples were scanned with a variable pixel size between 12 and 14 μm. Additional zoom scan was performed in order to not physically cut sample but allow a more detailed reconstruction of the internal bony structures (such as OSL). Therefore, the entire OSL of cochlea 3 was scanned at 4.5-μm nominal resolution (Fig. 2), while the basal and middle turns and apex of cochleae 1 and 2 were scanned at voxel sizes of 2.3 to 2.5 μm, respectively, depending on the available field of view.

### Measuring the OSL Throughout the Cochlea

**Fig. 3:**
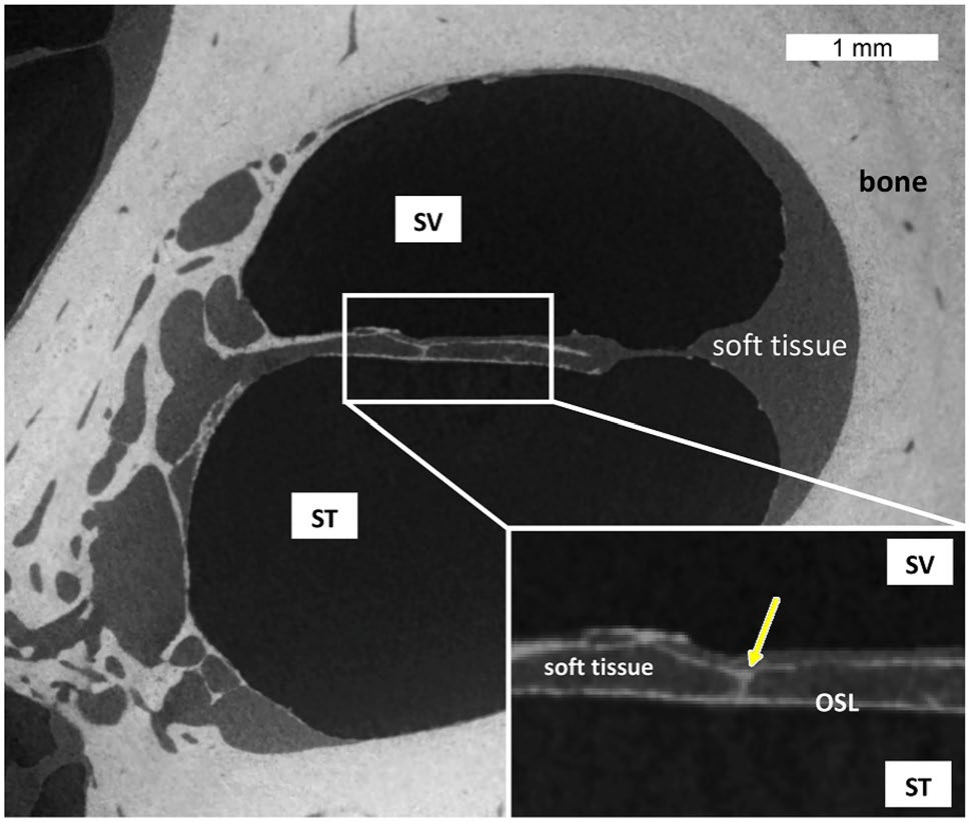
MicroCT cross section of the cochlea basal turn demonstrating the OSL and the difference of densities between soft tissue and bone. Yellow arrow points to one pillar that could be seen on the image inset, connecting both OSL plates. SV, scala vestibuli; ST, scala tympani

For the 3D reconstructions of OSL and the individual plates, the medical image analysis software Amira (Thermo Fisher Scientific, Waltham, MA, USA) was used. Selected global thresholds were used to create the 3D images in all datasets. To measure the OSL throughout the cochlea, we applied an interactive threshold to binarize the OSL bone that shows a different gray level compared to soft tissue in the microCT sections (Fig. 3). The threshold was determined through a collaborative effort between an imaging expert and a radiologist with extensive experience in cochlear imaging. Since the technique used to scan the middle turn and the apex of cochlea 1 was not specific to visualize the OSL, some artifacts were found on the raw image that were visually interfering on the final mesh creation. Because these artifacts could potentially interfere on the porosity measurement, they were removed using image software Meshmixer (Autodesk Inc., San Francisco, CA, USA). In addition, the middle turn and the apex were manually separated using the editing tool. The exact location for the cut was estimated using the raw image loaded at the same time as the segmented OSL. The segmented OSL voxels were added to a label, which was subsequently used as a mask to isolate the labeled anatomy in the original.tiff images, creating the 3D mesh and STL file. Additional segmentations of the OSL plates (tympanic and vestibular) were performed using the same criterion as above on higher resolution datasets to allow evaluation of porosity.

The segmented images were then further analyzed in Mimics (Materialise NV, Leuven, Belgium). On the mesh object, the spiral ganglion (SG) and the inner and outer walls of the OSL were contoured using the spline function. The middle axis was set by considering the helicotrema as the center point of the cochlea and using it as a reference axis. Following contouring, the measurements were conducted on reconstructed splines in a custom script (Python 3.9.0). The OSL width was calculated from apex to base as the distance between the inner (modiolus) and the outer wall (between the lateral end of the OSL) along the reference axis. Within each partition, the mean width and the standard deviation (SD) were also calculated.

### Porosity Measurement

Numeric calculations for porosity measurements were done in Blender (open source 3D computer graphics software version 3.1.2). Each plate (tympanic, vestibular) in the selected regions of interest of the cochlea (basal turn, middle turn and apex) was imported into workspace as an STL file, remeshed and decimated (using remesh and decimate modifiers) prior to measurement in order to reduce the computation cost and ensure manifold structures. In order to calculate the filled volume of the plate mesh needed for porosity calculation, the original mesh was shrink-wrapped using an encircling object that follows its shape. Lastly, the volumes of the original and filled meshes were estimated using the 3D print plug-in (volume statistics), and the porosity was calculated with the equation:

$$p=\frac{{V}_{f}-{V}_{o}}{{V}_{f}}\bullet 100 \%$$

where *V*_*o*_ denotes the original (porous) volume and *V*_*f*_ its filled (nonporous) counterpart.

### Thickness and Width Measurement

Thickness of the OSL is defined as the transverse height between the VP and TP [6]. To study the OSL thickness, microCT cross-section images (14-, 13-, and 4.5-µm voxel size) were used and three points of the OSL were chosen for measurements. These points correspond to the lateral end (where the CP is inserted), the middle of the OSL, and the medial portion of the OSL (close to the SG). When calculating the width of the OSL, care was taken not to include portions of the SG in the measurements. The segmented cochlea was used, and four anatomical dissection planes, having the helicotrema as the planes’ converging point, were placed at 45^0^ to each other to facilitate angular identification and measurement. Assessment was done from the medial portion up to the end of the HO, throughout all the cochlea spirals. The mean and standard deviation (SD) of the three specimens were calculated for both thickness and width.

## Results

### OSL Anatomy

The OSL is a bony structure suspended inside the cochlea like a “cable-stayed” bridge. Medially, it is connected to the modiolus with rigid bony “cables”. Laterally is connected to the collagen fibers that run along the bridge and basilar membrane, then to the spiral ligament as shown in Raufer et al. [5] with polarized microscopy to visualize collagen fibers. It contains nerve fibers running from the organ of Corti to the SG, seen on the microCT (at 13- and 4.5-µm voxel size resolution) and image as soft density material inside the OSL (Fig. 3).

**Fig. 4:**
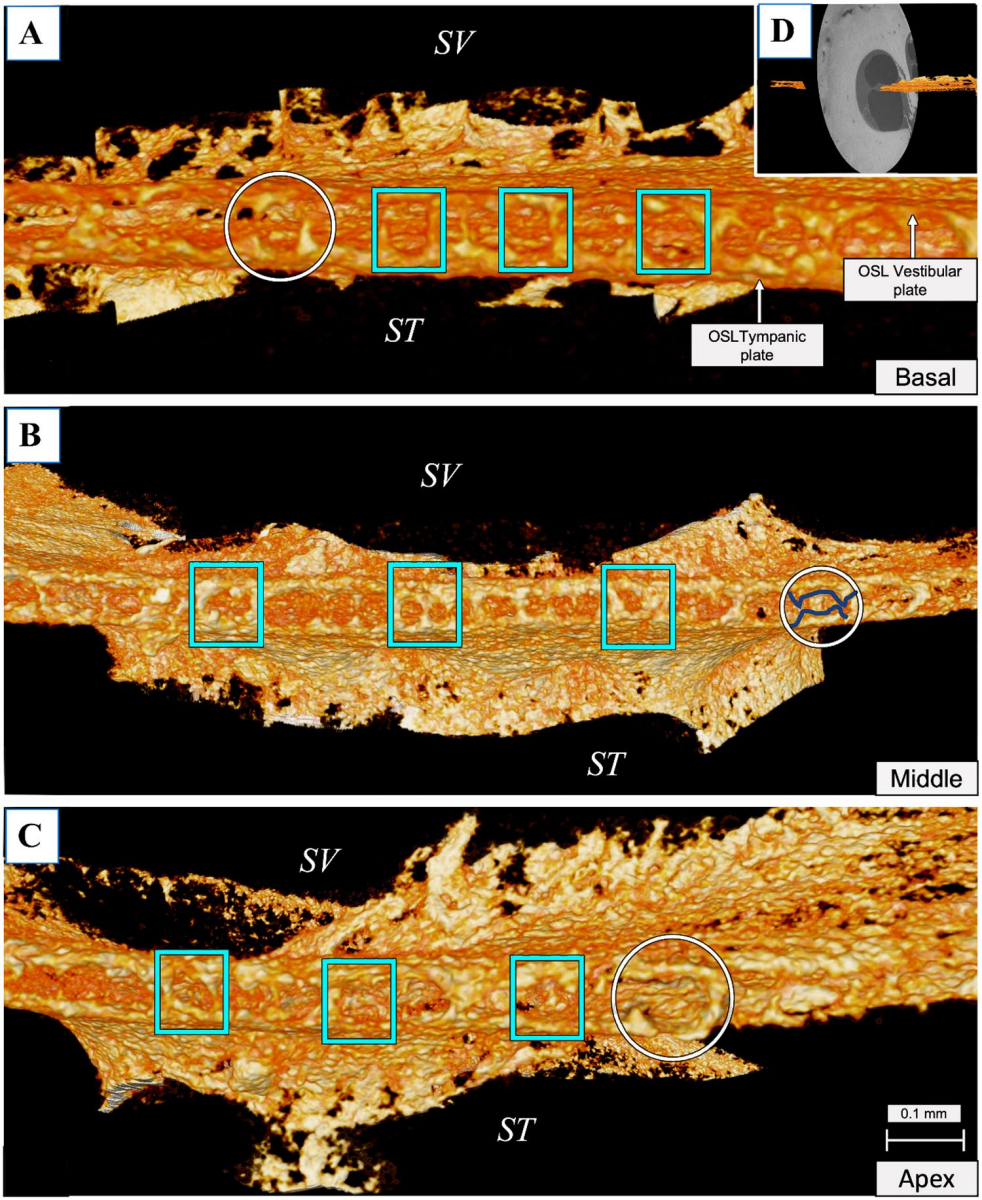
Mesh reconstruction of the habenular openings (blue squares) seen at the edge of the OSL opposite to modiolus andtangential to the habenula perforata in **A** the basal turn, **B** the mid-turn; and **C** the apex using 2.4-μm voxel size zoom CT scans. The circles show the “claw” aspect (outlined in dark blue on the middle turn) of the unclosed HO. **D** shows the orientation and positioning of the OSL in the raw CT image. SV, scala vestibuli; ST, scala tympani

Analyzing the OSL plates, it appears that the structure behaves as a lace cover for the nerve fibers. Where the tympanic plate (TP) joins the vestibular plate (VP), it forms the habenular openings (HO) (Fig. 4). The HO are arranged in intervals and are the entry point for the soft tissue fibers that form the bridge region to the OSL. These fibers will gather on the spiral ganglion and will later form the cochlear nerve. When the bones of the plates are not completely connected by bony pillars, a claw-like structure (such as incomplete bony pillars) is observed, as shown in Fig. 4 in the circles and outlined in dark blue on the middle turn. The HO are evenly distributed throughout the OSL, including the apex (Fig. 4).

**Fig. 5:**
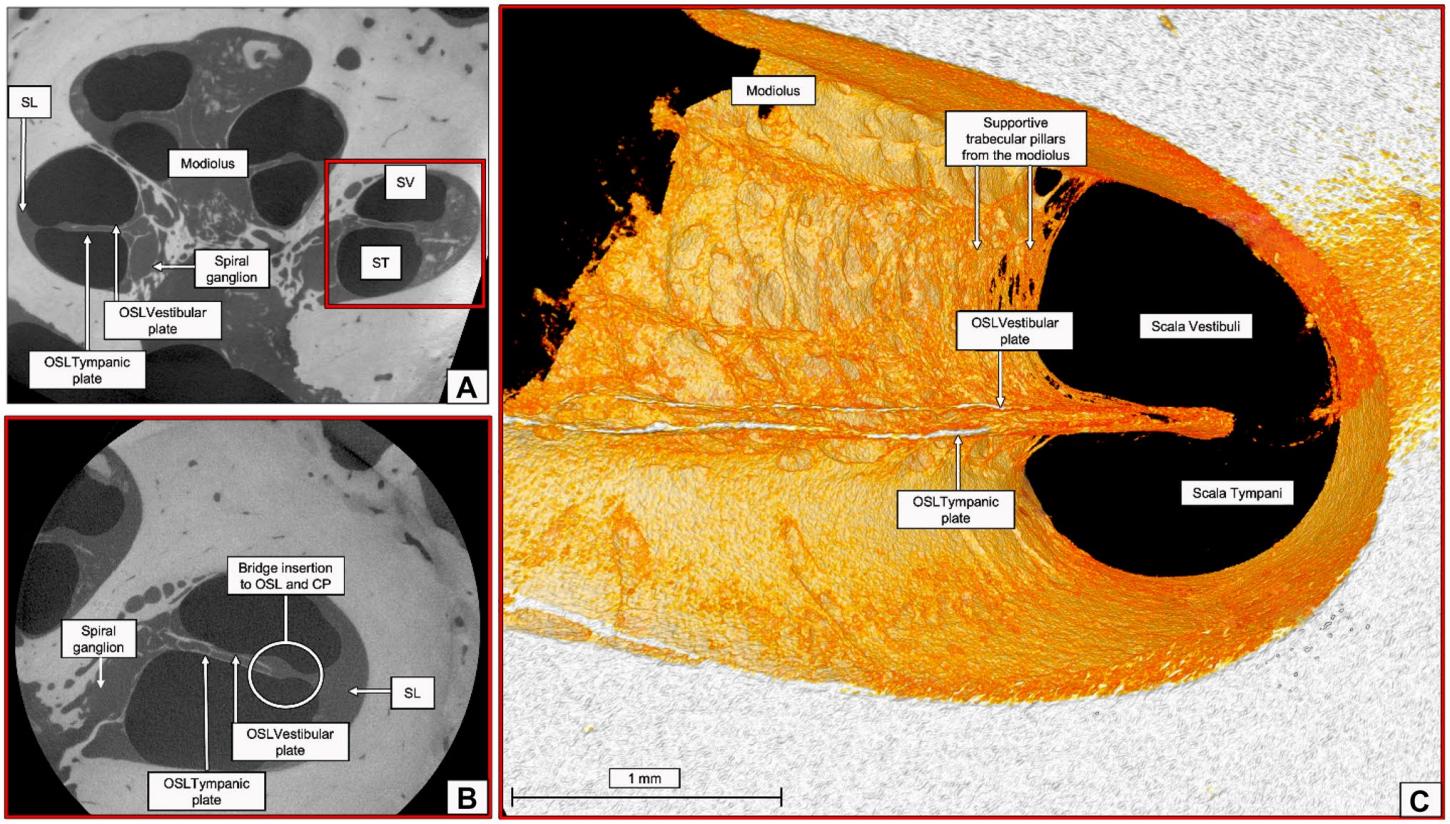
**A** 4.5-µm voxel size microCT of cochlea 3. Red square represents the area of the zoom scan. **B** At 2.3-µm voxel size, the OSL is visualized, and its plates are individually identified, as well as the insertion point of the bridge and the cochlear partition region, described by Raufer et al. [5, 6]. **C** Mesh reconstruction of **B** raw image emphasizing the supportive trabecular bone of the modiolus wall and the individual OSL plates. SL, spiral ligament

Using the 4.5-µm nominal resolution, the modiolus was analyzed and reconstructed in 3D (Fig. 5). By suppressing the soft tissue density material during the mesh reconstruction, it was possible to emphasize the bony structures. Detailed results regarding this cochlea are available here [22]. The medial wall (modiolus) of the tympanic pillar presents as columns of thin bone with a cribriform appearance. All three specimens showed the same characteristics.

### Beyond the Habenular Openings and the Bony Pillars 

**Fig. 6:**
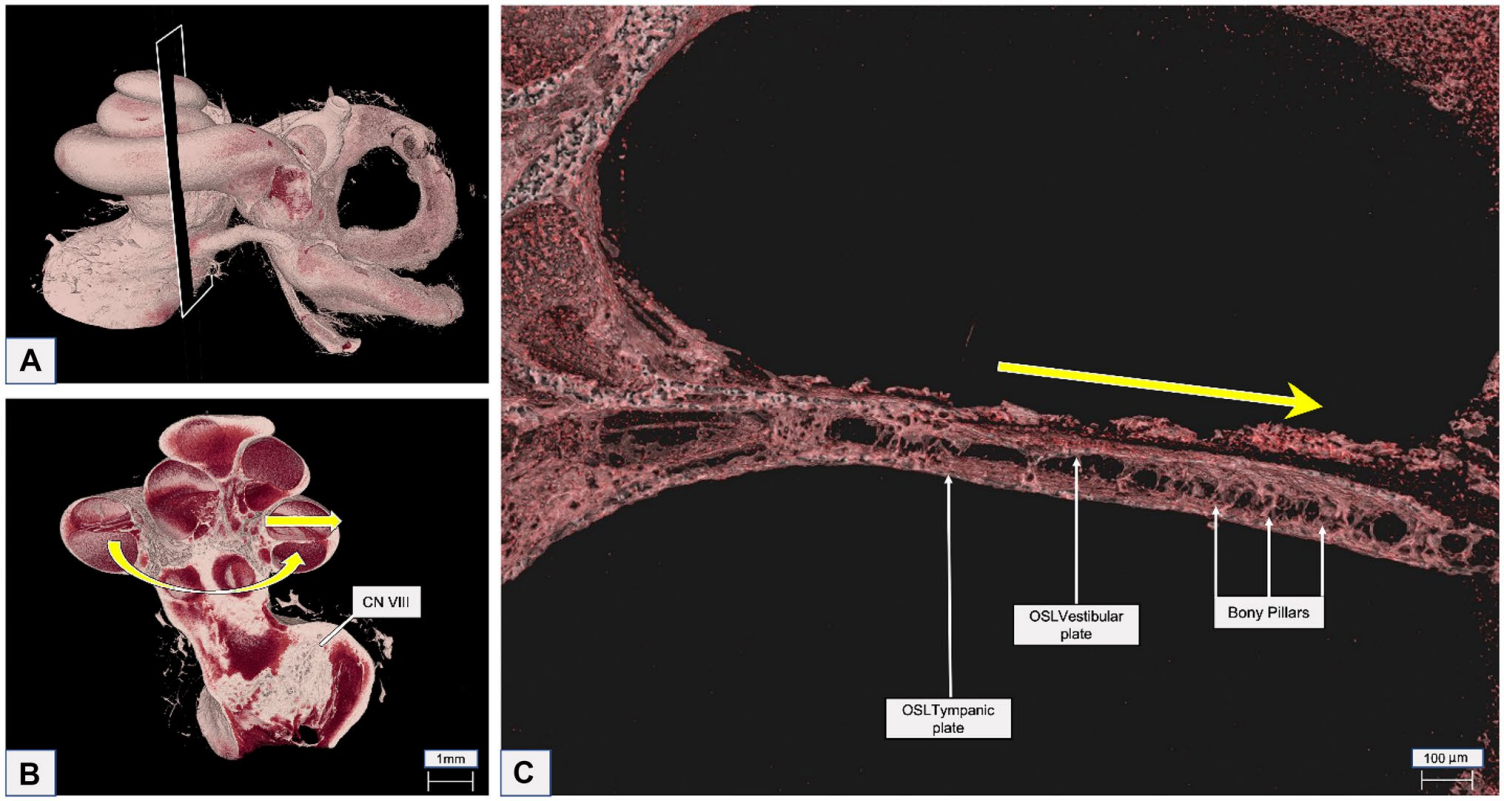
**A** A coronal cut on the full inner ear segmentation at 13-µm voxel size was made, and the OSL and the inner ear nerves were exposed. **B** The VIII cranial nerve (CN VIII) can be seen as light pink color. Curved arrow shows the longitudinal direction of the OSL, and straight arrow shows the radial direction of the OSL. **C** Using the 2.3-µm voxel size, resolution, and radial direction from (**B**), it was possible to demonstrate the bony pillars of the OSL and the OSL vestibular and tympanic plates. OSL, osseous spiral lamina

**Fig. 7:**
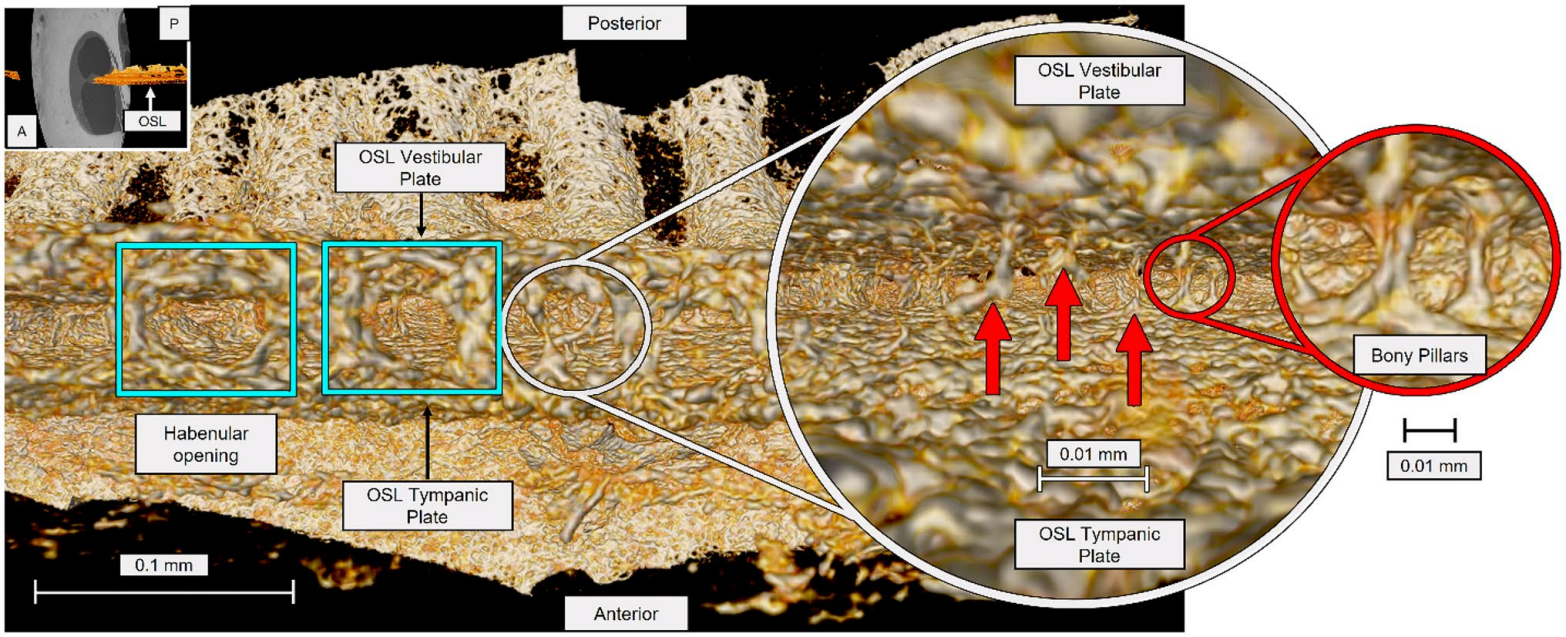
Mesh reconstruction of the inside of the human OSL using 2.3-µm resolution. Anterior view, tangential to the habenula perforata, of the OSL demonstrating the HO (blue squares). Entering through the HO (small white circle), inside the OSL, the vestibular and tympanic plates of the OSL are seen. The bony pillars (red arrows) are demonstrated in detail on the zoomed red circle

Visualizing the HO was possible using 2.3- to 2.5-µm reconstructed 3D mesh images. The sparse distribution of the pillars on the inside of the OSL and throughout its length was clearly evident. Inside the OSL, between the vestibular and tympanic plates, are BP that are randomly distributed in both longitudinal and radial directions (Fig. 6). The BP are present in larger numbers closer to the SG, and they appear to connect the VP to the TP on the inside, granting the OSL a cave-like aspect. Some pillars do not merge together and resemble stalactites and stalagmites emerging from the VP and TP, respectively. The middle of the OSL, where the plates are more porous, appears to have few BP, whereas the closer it is to the SG, the more BP there are. Near the SG, the thickness of the pillars appears to increase as well as their numbers. From the inside, it is possible to observe the pores of the plates and the back of the HO (Posterior) (Fig. 7). Combining the global view of the OSL plates with the local reconstruction of its interior at higher resolution, one can describe the OSL as a lace-like cover for the nerve fibers and hypothesize that the BP may have a support function for the OSL.

### OSL Morphology

**Fig. 8:**
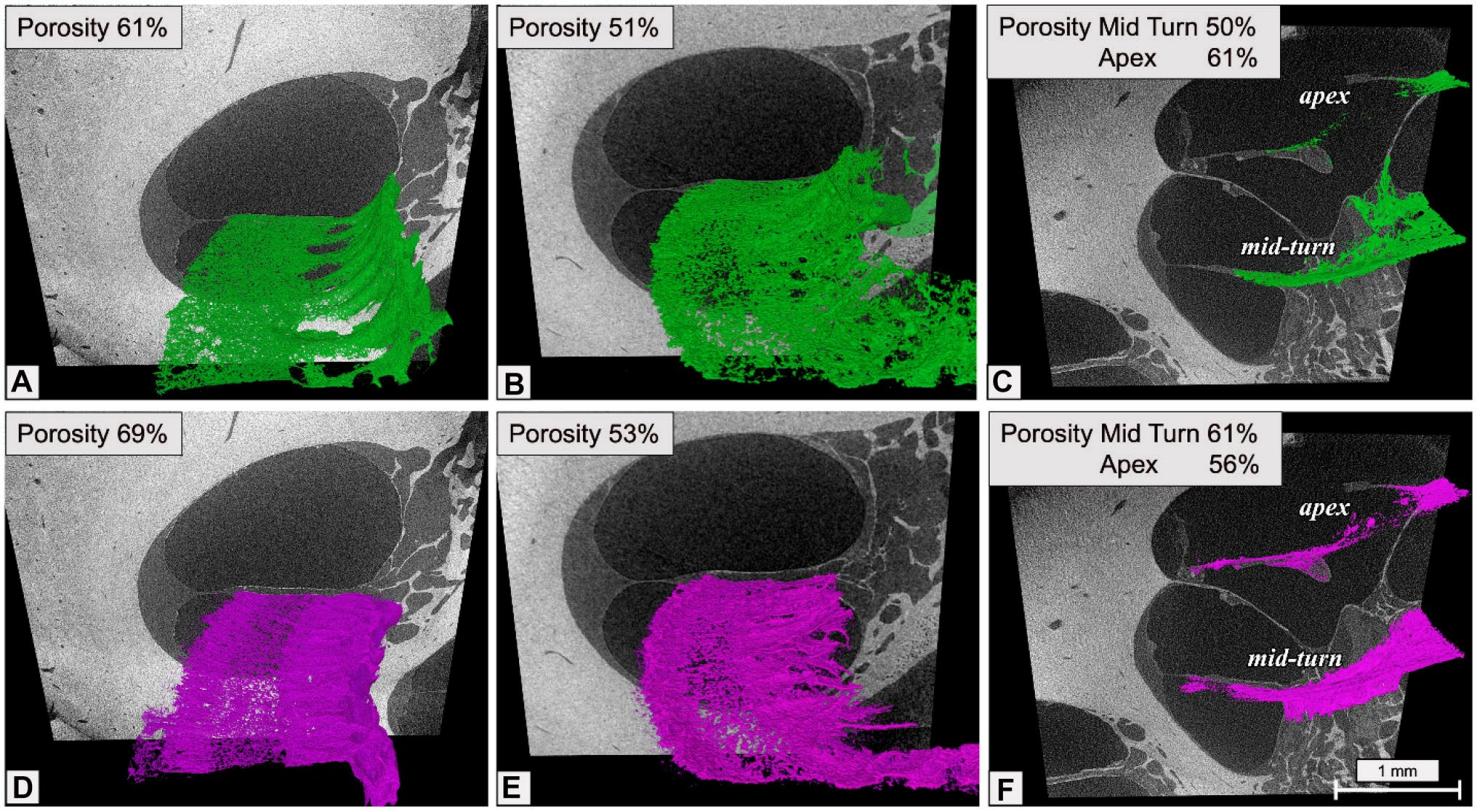
Individual segmentation of the basal turn of the osseous spiral lamina. **A** and **D** Cochlea 1 basal turn porosity results for the vestibular plate (green) and tympanic plate (purple), respectively. **B** and **E** Cochlea 2 basal turn porosity results for the vestibular plate (green) and tympanic plate (purple), respectively. **C** and **F** Cochlea 1 mid-turn and apex porosity results for the vestibular plate (green) and tympanic plate (purple)

**Fig. 9:**
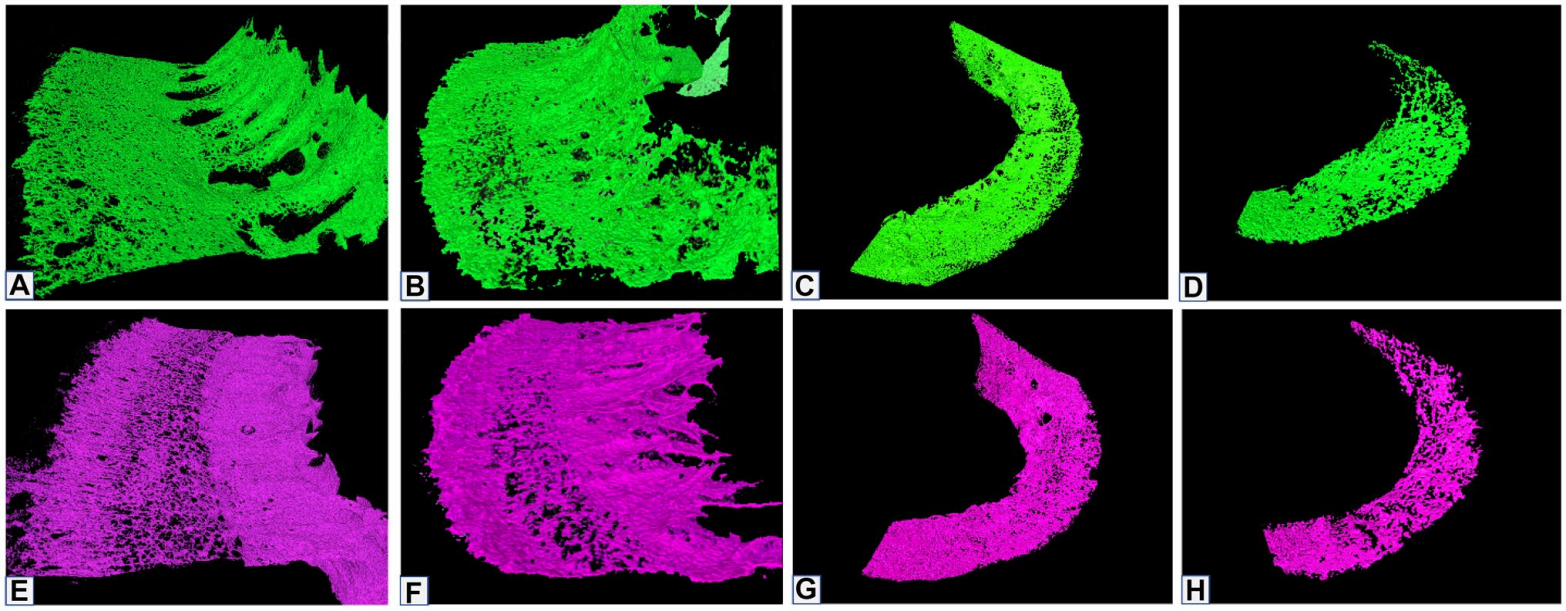
Porosity distribution at the basal turn, mid-turn and apex of the osseous spiral lamina. Vestibular plates are colored green (**A** to **D**), and tympanic plates are purple (**E** to **H**). **A** and **E** Basal turn cochlea 1; **B** and **F** basal turn cochlea 2; **C** and **G** mid-turn cochlea 1; **D** and **H** apex cochlea 1

The plates of the OSL were individually segmented at the level of the basal turn, middle turn, and apex for cochlea 1 and only the basal turn for cochlea 2. Both the TP and VP appeared to be of trabecular bone, giving the OSL a porous surface. The columns and channels formed by the merging of the TP and VP on the modiolus wall seem to be channel openings for the cochlear nerve fibers. The individual segmentation of TP and VP of cochleae 1 and 2 showed that the pores are distributed over the bone surfaces on both TP and VP. The TP (69% and 53% of porosity for cochlea 1 and cochlea 2, respectively) is more porous than the VP (61% and 51% of porosity) on the basal turn, and on the middle turn (TP, 61%; VP, 50% of porosity). On the other hand, the apex presented inverse values with TP, 56%, and VP, 61%, of porosity (Fig. 8). These values are at least 30 percentage points higher than those previously reported in the literature [6]. Figure 9 shows the distribution of the pores throughout both VP and TP throughout the basal turn, middle turn, and the apex. Pores are predominant in the middle portion on the basal turn, while the middle turn and the apex presented a more even distribution.

**Fig. 10:**
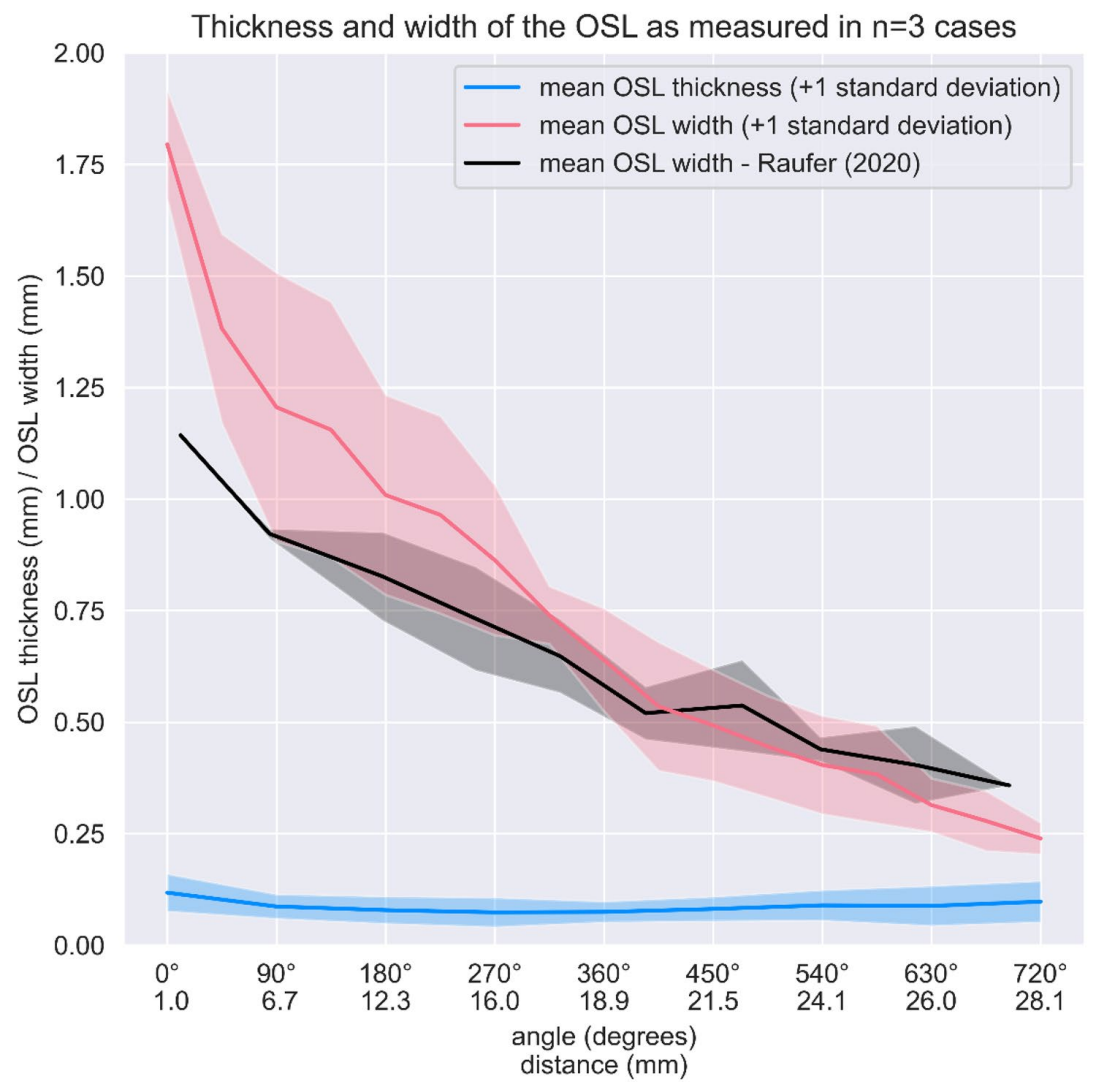
Mean and standard deviation of osseous spiral lamina (OSL) thickness and radial width. The expected decrease of the OSL width is shown as well as the constant thickness values throughout the spirals. Raufer’s [6] data was included to complement the analysis

The results of the thickness and width of OSL were calculated and compared to previous data obtained by Raufer et al. [6] (Fig. 10). Whereas the thickness of the OSL is constant at around 0.1 mm, with a minimum about 20% lower around halfway into the cochlea (i.e., at 360 degrees), the width of the OSL decreases from approximately 1.8 mm at the base to 0.2 mm at the apex (i.e., at 720 degrees).

## Discussion

The cochlea is a small, spiral bone structure in the inner ear that plays a critical role in hearing. It contains three types of bones: (1) the petrous portion of the temporal bone, which is the trabecular bone that houses the cochlea; (2) the modiolus, which is the central conical axis of trabecular bone; and (3) the osseous spiral lamina (OSL), which is a thin layer of bone composed of two plates of compact bone that protrudes from the modiolus and separates part of the two fluid-filled canals that run through the cochlea, the scala vestibuli, and the scala tympani, which, like the canals, makes two and three-quarter turns around the modiolus [23, 24].

The bony structures of the inner ear were superficially described in early anatomical studies, but imaging demonstration has only been possible more recently [1, 2, 16]. Anatomical descriptions of the bony inner ear are usually restricted to the modiolus [16, 25] and use histopathological techniques. Although histopathology remains the gold standard for cytological evaluation, the specimen preparation process involves the physical destruction of the specimen and creates typical anisotropic resolution (0.5 × 0.5 × 100 µm) if translated into 3D [26–28].

Surrounded by the dense cochlear bone, under around 2.5-µm resolution microCT, a trabecular looking modiolus stands in the middle of the cochlea, projecting itself to form the two bony plates that determine the shape of the OSL. Although the OSL consists of two plates of compact bone, the porous nature of these plates and the presence of numerous bony pillars make it more similar as a whole to a trabecular bone. In fact, trabecular bone is by definition a hierarchical, spongy, and porous lattice structure that provides the framework for the soft, highly cellular bone marrow filling the intertrabecular spaces. At a microstructural scale, trabecular bone architecture is organized to optimize load transfer [29]. Similarly, the OSL system as a whole can be thought of as trabecular bone, with two individual plates connected by bony pillars on the inside and the habenular openings at its end. The OSL plates have a lace-like appearance enfolding the fibers that will later form the cochlear nerve. The radial middle portion of the basal turn of the OSL (both plates) has a more porous appearance than its edges, similarly to Raufer’s description [6]. This surface reduction is congruent with the sparse distribution of the bony pillars in the middle of the OSL. Although habenular openings were described by Küçük et al. [15] as tunnels, internal inspection of the structure indicates that the openings do not behave like tunnels. On the contrary, they convey the impression of a cave and appear to have a structural support function, joining the plates together on the lateral end, at the insertion point of the bridge and limbus regions. The bony pillars could have a similar support function, keeping the plates together while providing stability that can have a potentially significant influence on the spread of excitation during its vibration. However, more investigation on this possibility is needed.

The lace-like appearance of the OSL plates turns out to be a common characteristic, and it was observed in the measured specimens. Porosity analysis demonstrated a considerable higher percentage of pores on both vestibular plate VP and tympanic plate TP on the basal turn if compared to previous study [6]. Unlike the work of Raufer et al. [6], which was limited to considering different portions of the basal turn and suprabasal turn (between 1 and 12 mm of longitudinal distance from the base), in this study, we also calculated porosity relative to the middle turn and the apex. In these cases, we found a constant presence of porosity in both TP (mid, 61%; apex, 56%) and VP (mid, 50%; apex, 61%). Moreover, while the TP appears to be more porous than VP in the basal and middle turns, although this difference is less pronounced than in previous descriptions [6], in the apex, we find more porosity in VP than in TP (61% vs. 56%). With regard to our study results, we believe that the volumetric reconstructions used in our measurements provide a more realistic depiction of the OSL anatomy. Since 3D investigation provides more information than conventional 2D studies, this technique seems to be more suitable for demonstrating the porosity of the structure. The difference in methodology can also be responsible of the main differences found in the analysis of OSL width compared to previous studies as described in Fig. 10. The major discrepancy was found at the basal turn where, besides the use of different methodologies, both studies lack in samples group size (our pilot study *n* = 2; previous study *n* = 1). Further investigation will have to be carried out to provide reliable statistics especially on distances closer to the base. The combination of a highly porous trabecular bone and the fluid filled cochlea creates a new approach for hearing mechanical studies. Evidently, future kinetic cochlea studies must consider the OSL and the cochlear partition as one mobile structure, the differences in porosity from base to apex, and the effects of the fluid dynamics in their vibration analysis calculations. This modeling work can help support recent theories regarding the influence of different material properties on the motion of the cochlear partition and contribute to the understanding of low-frequency and bone conductive hearing [30]. As a result, it will be useful in the development of clinical interventions for the preservation of hearing.

Another possible clinical consequence of the OSL’s porosity is that it can potentially play a role in manufacture of new implant technology, direct inner ear drug delivery, and stem cell delivery. Secondary to hair cell loss, spiral ganglion’s neuron degeneration is one of the major causes of sensorineural hearing loss. The use of perilymph as carrier for inner ear drug delivery is common, but it is inefficient due to the difficulty in reaching stable therapeutic concentrations throughout the cochlea [31–37]. The spiral ganglion’s neurons are located in Rosenthal’s canal which runs along the OSL, and there is increasing evidence of their permeability to small molecules and neurotrophic genes [38–42]. The difficulty of drugs in crossing the blood–labyrinth barrier has been reported [33, 43, 44]. In the future, drug-eluted cochlear implants could potentially take advantage of the OSL’s porosity to enable drug distribution directly to the spiral ganglion [45].

Geometrical reconstruction of the OSL revealed a cantilever structure that should be considered in future mathematical models for studying hearing mechanics. Although it should be noted that the voxel size used in this pilot study may be considered relatively large to obtain accurate measurements, especially in the OSL thickness range, the thickness and width we measured are consistent with previous studies [6, 7]. However, in contrast to earlier findings [6], our porosity measurements demonstrated the presence of pores in both VP and TP (up to 69%), as well as random bony pillars distribution throughout the cochlea in both radial and longitudinal directions. Additionally, it was possible to observe the habenular openings through a new perspective; rather than the previously described “tunnel”, our study showed a “cave”. Furthermore, the connection between the cochlear partition and the OSL that we have demonstrated in 3D should encourage others to consider that they possess a “one flexible structure” morphology. In this article, we present three of four factors (i.e., geometry) to be considered in future studies of hearing mechanics: the width, thickness, and stiffness (porosity) of the OSL. The inter-plate bony pillars (including its thickness and related stiffness) should also be considered as a factor and added to the mathematical models. Gathering information on these variables by applying 3D methodology described here to a larger sample size will provide a better understanding of hearing mechanics.

## Conclusion

A combination of surgical dissection techniques with microCT allowed, for the first time, an isotropic volumetric study of the human OSL. Our study demonstrated the porosity of both plates (VP and TP) and the presence of bony pillars in the middle of the OSL. It also enabled a flight through the habenular openings and throughout the whole cochlea. The microCT technique emerges as a valuable imaging option in hearing research, providing a detailed anatomical 3D-reconstruction of the ear. Furthermore, visualizing the anatomical details of an intact cochlea at the micrometer level, with high resolution, and in 3D provides valuable new information for application to future anatomical, physiological, and mathematical studies.

## Abbreviations

A: Anterior
BP: Bony pillars
CP: Cochlear partition
HO: Habenular openings
OSL: Osseous spiral lamina
P: Posterior
SD: Standard deviation
SG: Spiral ganglion
SL: Spiral ligament
ST: Scala tympani
SV: Scala vestibuli
TP: Tympanic plate
VP: Vestibular plate
